# Model-based cost-effectiveness analyses comparing combinations of urate lowering therapy and anti-inflammatory treatment in gout patients

**DOI:** 10.1371/journal.pone.0261940

**Published:** 2022-01-28

**Authors:** Celine Johanna van de Laar, Carly A. Janssen, Matthijs Janssen, Martijn A. H. Oude Voshaar, Maiwenn J. AL, Mart A. F. J. van de Laar

**Affiliations:** 1 Transparency in Healthcare BV, Hengelo, the Netherlands; 2 Department of Psychology, Health & Technology, University of Twente, Enschede, The Netherlands; 3 Department of Rheumatology, VieCuri Medical Center, Venlo, The Netherlands; 4 Institute for Medical Technology Assessment, Erasmus School of Health Policy & Management, Erasmus University Rotterdam, Rotterdam, The Netherlands; Haute Autorite de sante, FRANCE

## Abstract

**Objectives:**

To assess the cost-effectiveness of various combinations of urate lowering therapy (ULT) and anti-inflammatory treatment in the management of newly diagnosed gout patients, from the Dutch societal perspective.

**Methods:**

A probabilistic patient-level simulation estimating costs and quality-adjusted life years (QALYs) comparing gout and hyperuricemia treatment strategies was performed. ULT options febuxostat, allopurinol and no ULT were considered. Flare treatments naproxen, colchicine, prednisone, and anakinra were considered. A Markov Model was constructed to simulate gout disease. Health states were no flare, and severe pain, mild pain, moderate pain, or no pain in the presence of a flare. Model input was derived from patient level clinical trial data, meta-analyses or from previously published health-economic evaluations. The results of probabilistic sensitivity analyses were presented using incremental cost-effectiveness ratios (ICERs), and summarized using cost-effectiveness acceptability curves (CEACs). Scenario analyses were performed.

**Results:**

The ICER for allopurinol versus no ULT was €1,381, when combined with naproxen. Febuxostat yielded the highest utility, but also the highest costs (€4,385 vs. €4,063 for allopurinol), resulting in an ICER of €25,173 when compared to allopurinol. No ULT was not cost-effective, yielding the lowest utility. For the gout flare medications, comparable effects on utility were achieved. Combined with febuxostat, naproxen was the cheapest option (€4,404), and anakinra the most expensive (€4,651). The ICER of anakinra compared to naproxen was €818,504. Colchicine and prednisone were dominated by naproxen.

**Conclusion:**

Allopurinol and febuxostat were both cost-effective compared to No ULT. Febuxostat was cost-effective in comparison with allopurinol at higher willingness-to-pay thresholds. For treating gout flares, colchicine, naproxen and prednisone offered comparable health economic implications, although naproxen was the favoured option.

## Introduction

Gout is an inflammatory response to the presence of hyperuricemia induced monosodium urate (MSU) crystals within the synovial fluid of joints and tissues. It is the most common cause of inflammatory arthritis in men, and reports have shown the burden of gout to be rising [1]. Gout attacks are characterized by rapid onset of severe pain and may have a considerable impact on patient’s ability to work and function in other social roles [2, 3]. Typically, gout attacks resolve within 5–7 days with effective anti-inflammatory treatment. Recurrent attacks, and the development of chronic, inflammatory gout, may be prevented by effective urate lowering therapy (ULT) aimed at lowering serum urate (SUA) levels below the saturation point for crystal formation [4]. In light of the increasing burden of gout, the importance of optimizing treatment and management of gout at various levels, including patient, community and national, is emphasized.

Various safe and effective anti-inflammatory therapies are available for the treatment of both gout attacks and hyperuricemia. Allopurinol and febuxostat are currently recommended first-line ULT agents [5]. Colchicine, non-steroidal anti-inflammatory drugs (NSAIDs) and glucocorticosteroids are all first-line treatment options for treating gout flares [6]. Besides these traditional synthetic medications, targeted biological medications, in particular interleukin-1 (IL-1) inhibitors, including anakinra and canakinumab, have been investigated in recent clinical trials for treating gout flares [7, 8]. IL-1 inhibition is currently recommended as a second-line treatment option for managing gout flares [5, 6].

Due to the high and increasing prevalence of gout, and the introduction of novel treatment options such as the relatively expensive IL-1 inhibitors, health economic implications are important to consider when deciding on optimal treatment approaches for patients with this disease [9]. Health economic decision models that have thus far been developed to support such decision making are mainly concerned with the comparison of various ULTs [10–12]. Although some models do account for gout flares by assigning disutilities, the effects and costs of anti-inflammatory treatments are not explicitly considered in addition to or instead of ULT. However, with the introduction of new, more costly and potentially more effective treatments for treating gout flares, simultaneously evaluating outcomes of ULT and anti-inflammatory medications becomes more relevant. In the present study we introduce a new modelling framework for gout, in which the costs and effects of treatment strategies with continuous ULT and anti-inflammatory medications for gout flare can be assessed in newly diagnosed patients. This model compares the costs and effects of various commonly recommended and administered ULT and anti-inflammatory treatments from a Dutch societal perspective.

## Methods

For this study, model-based cost-effectiveness analysis was performed from the societal perspective, in which first-line ULT agents for hyperuricemia (i.e. allopurinol, febuxostat, no ULT), as well as first-line (i.e. colchicine, naproxen and prednisone) and second-line (i.e. anakinra) treatment options for gout flares were compared. Medication costs, other healthcare costs and productivity loss were included. The Dutch Willingness To Pay (WTP) threshold is not strictly defined and can be calculated based on disease burden. The Consolidated Health Economic Evaluation Reporting Standards (CHEERS) statement was followed in reporting the results of this cost-effectiveness analysis (See **S1 File**).

### Markov model

**Fig 1:**
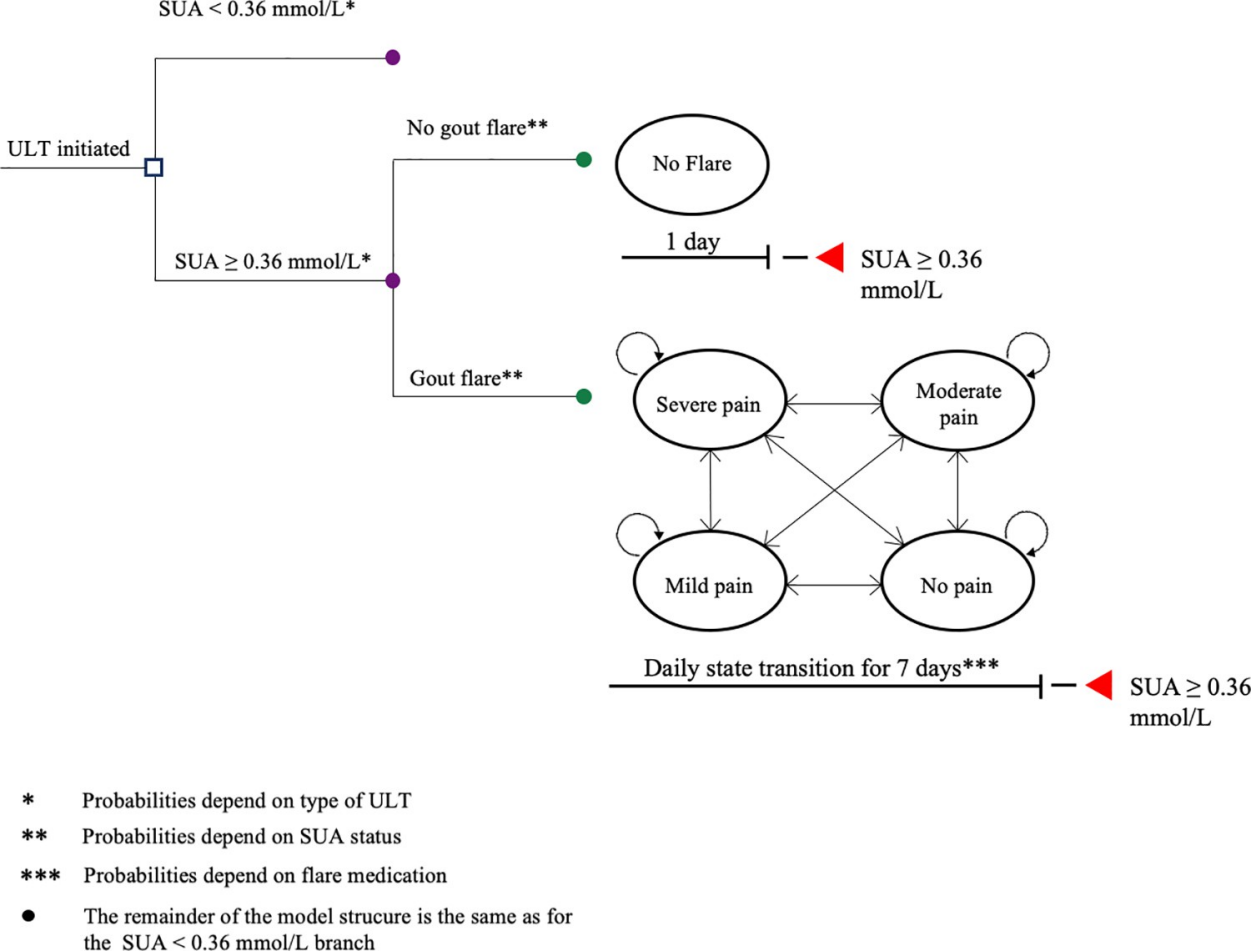
Model structure. ULT, urate lowering therapy; SUA, serum urate. * Probabilities depend on type of ULT; ** Probabilities depend on SUA status; *** Probabilities depend on gout flare medication; • The remainder of the model structure is the same as for the *SUA < 0*.*36 mmol/L*-branch.

A Markov Model (TreeAge™) was developed to simulate and compare outcomes of various ULT and anti-inflammatory treatment combinations for hyperuricemia and gout flares, respectively (Fig 1). For the present study the time horizon was one year with a cycle length of one day. Due to the nature of the available data for anakinra and the absence of long-term effects of gout in the model a much longer horizon would not be appropriate. Running a longer-term model with the lack of long-term data would lead to serious omissions. The model considered the one-year course of newly diagnosed gout patients, receiving treatment for their gout flare, and who initiated ULT while experiencing a gout flare, reflecting a care path commonly applied for these gout patients in clinical practice. Upon entry in the model, patients were assigned a fixed dose of ULT, with either allopurinol (at 200 mg or 300 mg), or febuxostat 80 mg, or no ULT, based upon available data from the literature. The probability that the patient achieved the SUA target, defined as achieving a SUA level < 0.36 mmol/L, depended on the specific ULT. Patients were not able to switch after this first division had been made, thus, they could not go from SUA < 0.36 mmol/L to any other branche for the one-year duration of this model. After it had been determined if a simulated patient would have SUA levels on target (< 0.36 mmol/L) or not on target (≥ 0.36 mmol/L) for the duration of the simulation, patients experienced a daily risk of having a gout flare. Patients with SUA levels not on target (in the SUA ≥ 0.36 mmol/L branch) had a higher daily flare risk than patients in the on-target branch (SUA < 0.36 mmol/L). Patients in both branches were able to experience flares. Patients that did not reach the target did experience more and more frequent flares in this model. When a gout flare was triggered, patients transitioned for seven days between four mutually exclusive pain states (i.e. no pain, mild pain, moderate pain, severe pain) according to transition probabilities defined for each gout flare treatment option. Treatment options included in the model for gout flares were colchicine, naproxen, prednisone and anakinra. Dosages were in line with the dosages as used in the clinical studies used as data sources [8, 13]. No switching between ULT drugs, or medication for gout flares, were allowed in the model during the time horizon. Costs and quality-adjusted life years (QALYs) over one year were recorded for all strategies.

### Analyses

We employed probability sensitivity analyses (PSA) with 2000 x 200 model replications to take uncertainty around the point estimates of the model parameters into account. The results were summarized using cost-effectiveness acceptability curves (CEACs). Costs were discounted at 4%, utility was discounted at 1.5%, in concordance with the Dutch costing manual [14]. The WTP thresholds in the Netherlands are not explicitly set, but lie between €10,000 and €80,000 per QALY [15].

### Model inputs

All parameters used as input for the model, as well as the data source from which they were estimated, are listed in **Table 1**. Transition matrices were used in the model between pain states, these can be found in the **S1 Table**. Uncertainty regarding elements of the various transition matrices were expressed using Dirichlet distributions. For other input parameters, various distributions were fitted to the observed data. Chi-squared and Anderson Darling fit statistics were used to evaluate goodness of fit.

**Table 1:** Model inputs.

Parameter	Point estimate	Probability distribution	Source
**Probability SUA < 0.36 mmol/L**			
Allopurinol 200 mg	0.457	Beta (μ: 0.455, σ: 0.045)	[30]
Allopurinol 300 mg	0.480	Beta (μ: 0.478, σ: 0.027)	[31–33]
Febuxostat 80 mg	0.729	Beta (μ: 0.730, σ: 0.013)	[31, 33–36]
No ULT	0.000	n/a	[37]
**Daily flare probability**			
SUA on target with ULT	0.000716	Beta (μ: 0.999, σ: 0.002)	[12]
SUA not on target with ULT	0.001222	Beta (μ: 0.998, σ: 0.002)	[12]
SUA not on target with no ULT	0.001637	Beta (μ: 0.998, σ: 0.002)	[12]
**Quality adjusted life days**			
No pain	0.86	Beta (α: 16.325, β: 3.076)	[8]
Mild pain	0.77	Beta (α: 10.942, β: 3.177)	[8]
Moderate pain	0.70	Beta (α: 12.696, β: 5.329)	[8]
Severe pain	0.61	Beta (α: 19.817, β: 12.877)	[8]
**Daily other costs (in €s)**			
No pain	19.95	Exp/g (λ: 1.749 /α: 0.317, β: 3.623)^1^	[8, 14]
Mild pain	32.39	Exp/g (λ: 1.081 /α: 0.493, β: 2.135)	[8, 14]
Moderate pain	58.08	Exp/g (λ: 0.599 /α: 0.434, β: 4.149)	[8, 14]
Severe pain	134.32	Exp/g (λ: 0.259 /α: 0.291, β: 10.769)	[8, 14]
**Daily drug costs (in €s)**			
Colchicine	0.61	n/a	[19]
Naproxen	0.21	n/a	[19]
Prednisone	0.26	n/a	[19]
Anakinra	33.4	n/a	[19]
Allopurinol	0.13	n/a	[19]
Febuxostat	1.03	n/a	[19]

ULT: Urate lowering therapy, SUA: serum urate.

^1^ λ-parameter refers to WPAI (exponential distrubition) and α and β refer to the ZoCo (gamma distribution).

### Efficacy of ULT

ULT success was defined as achieving SUA level < 0.36 mmol/L. This SUA target level is recommended by guidelines, supported by reports that have shown that SUA levels below the target level of 0.36 mmol/L are associated with a decreased risk for gout flares [6, 16]. To generate model input, a meta-analysis was performed of ULT clinical trials, in which achieving the SUA target of < 0.36 mmol/L was one of the endpoints. The indirect adjusted comparison method, using febuxostat 40 mg as the reference treatment was used to obtain efficacy estimates and associated standard errors adjusted for study specific factors [17]. For all the placebo arms in the meta-analysis, the percentage of patients achieving the target was zero percent. Therefore for all treatment strategies in which patients do not use ULT it was assumed that no patient achieved the SUA target.

### Flare probabilities

Daily flare probabilities were calculated from data derived from a previous health economic model by Jutkowitz et al. 2014 [12]. In that paper, annual flare probabilities were given for patients on ULT with controlled SUA (< 0.36 mmol/L), for patients on ULT with uncontrolled SUA (≥ 0.36 mmol/L), as well as annual flare probabilities for patients not on ULT, with uncontrolled SUA (≥ 0.36 mmol/L).

### Efficacy of flare treatment

Health states for patients experiencing a gout flare were defined using four pain states (i.e. no pain, mild pain, moderate pain, severe pain), derived from a 4-point pain rating scale that is commonly used as a primary endpoint in gout clinical trials. Inverse variance weighted pain transition probabilities and their standard errors for naproxen and prednisone were obtained by pooling seven day [8]. And 90 hour [13] follow up data from two clinical trials. For colchicine and anakinra, the probabilities were obtained from the seven days follow up data of a single trial [8]. To avoid empty cells in the transition matrices, due to data sparsity, a Bayesian approach was used in which a transition matrix with 0.5 for each cell (i.e. noninformative prior) was combined with the observed transition frequencies [18]. Dirichlet distributions were then fitted on the resulting posterior distribution of transition probabilities in the PSA.

### Utilities

Utility weights were estimated for each of the four pain related health states using data obtained from the study by Janssen et al. 2019 [8]. The values of utility for each pain state were calculated from the SF-6D. Since a Dutch tariff is unavailable, the SF-6D health stages were valued with the UK tariff.

### Costs

We included costs related to gout drug use, healthcare resource utilization and work productivity loss due to gout and other reasons, in euros using Dutch price indices. The costs per day for each pain state were determined for all cost variables. The medication costs for the appropriate dosages were derived from the official Dutch list prices [19], and the cost per hour of foregone labour was retrieved from a report by The Netherlands Organizaton for Scientific Research [20].

Healthcare resource utilization for each pain state was estimated using patient-reported questionnaires obtained during a gout clinical trial, wherein patients were asked to report the number of visits to general practitioners, outpatient clinics for specialized caregivers, paramedical caregivers, but also the amount of household care that was used, and diagnostic tests (i.e. echo, CT scan) undergone [8]. The costs for each of these items were obtained from the 2015 Dutch Costing manual and corrected to the appropriate costing year [14]. Outliers, defined as costs deviating more than three standard deviations from the mean, were removed from the healthcare utilization data.

### Scenario analyses

Scenario analyses were performed for gout patients who did not experience a gout flare at model entry, and for gout patients with severe gout. For the latter, the daily flare chances were successively increased.

## Results

As the model used in this study incorporated treatment options for gout flares and hyperuricemia, there were two types of medication (i.e. anti-inflammatory treatment and ULT) compared in this study, resulting in many possible combinations. This section will first focus on different ULT medications combined with naproxen. Hereafter, the cost-effectiveness PSA results of different gout flare medication combined with different ULT medication types, will be presented.

### PSA Results ULT medication with naproxen as anti-inflammatory treatment

**Table 2** shows the PSA results of three ULT medication types combined with naproxen. No ULT was the cheapest option at €4,031.19. Allopurinol yielded more utility at a slightly higher cost of €4,063.94. Compared with no ULT, allopurinol was cost-effective with an incremental cost-effectiveness ratio (ICER) of €1,381.27. The most expensive option was febuxostat (€4,385.40), also yielding the highest utility. Compared to allopurinol, febuxostat had an ICER of €25,173. At a WTP-threshold of €25,173, this would be considered cost-effective in The Netherlands.

**Table 2:** Probabilistic sensitivity analysis for urate lowering therapy combined with naproxen.

	Costs (€s)	Effects (QALY)	ΔC (€s)	ΔE (QALY)	ICER (Δ€/Δ QALY)
No ULT + Naproxen	4,031.19	0.78877	-	-	-
Allopurinol + Naproxen	4,063.94	0.81248	32.75	0.02371	1,381.27*
Febuxostat + Naproxen	4,385.40	0.82525	321.46	0.01277	25,173.06**

QALY = Quality adjusted life years; ICER = incremental cost-effectiveness ratio; ULT = urate lowering therapy

*ICER for allopurinol vs NO ULT

** ICER for febuxostat vs allopurinol.

**Fig 2:**
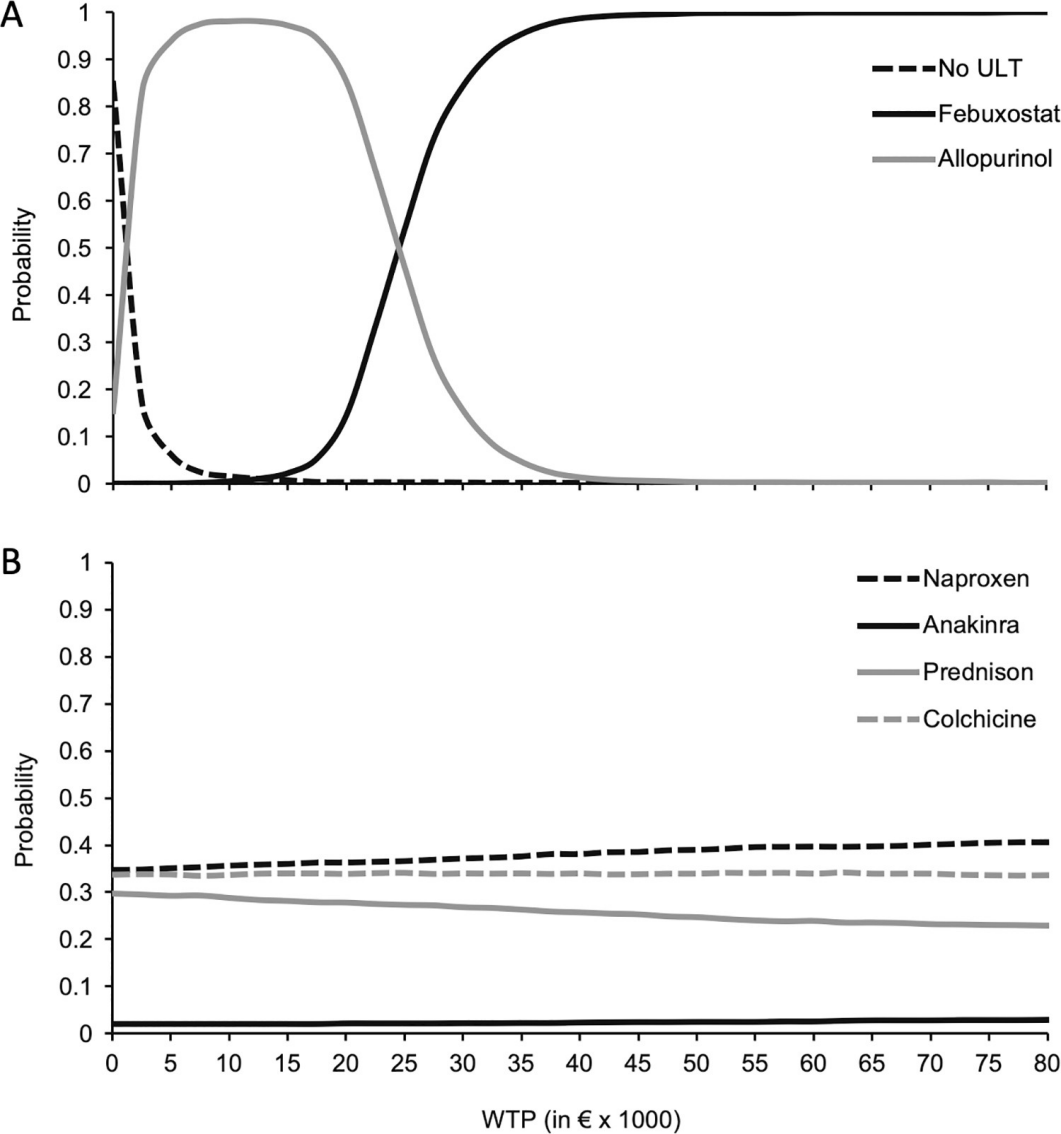
Cost-effectiveness acceptability curves (CEAC). ULT, urate lowering therapy; WTP, willingness-to-pay. Panel A displays the CEAC for different ULT combined with naproxen as the anti-inflammatory agent. Panel B displays the CEAC for different anti-inflammatory treatment options combined with febuxostat as the ULT.

Fig 2 displays the cost-effectiveness acceptability curves (CEACs). Overall, the differences in utility between acute flare medications (anakinra, colchicine, naproxen, and prednison) are very small. This is also reflected in the CEAC. The 0 point at the y-axis reflects the probability of each of the strategies being cost-saving, whereafter all points on the graph reflect the certainty with which we can say that the ICER is acceptable. The first panel (A) of Fig 2 shows that at WTP-thresholds below €25,173, allopurinol is the preferable option. At a WTP-threshold of approximately €25,000, there is a ~50% chance that allopurinol is cost-effective. At WTP-thresholds above €25,173, the probability with which febuxostat is cost-effective grows to ~100%, at a WTP of approximately €35,000. Results and implications were similar when combining the ULT medication with any of the other flare medications. The second panel of Fig 2(B) shows the CEAC of four strategies, febuxostat combined with the four anti-inflammatory medications. Over the full range of WTP threshold, febuxostat + naproxen has the highest probability of being cost-effective, ranging from 36% to 39%. Febuxostat combined with colchicine remains around 35% and febuxostat + prednisone ranges from 30% to 24%.

### PSA results comparing anti-inflammatory treatments with febuxostat as ULT

The different anti-inflammatory treatments had comparable effects on utility over 1 year when they were combined with febuxostat as ULT. PSA results showed that when naproxen was combined with febuxostat, patients accrued an estimated 0.81 QALYs over the course of 1 year. Larger differences between flare medication were seen in costs. Naproxen combined with febuxostat was the cheapest option at an estimated cost of €4,404,- per year. Colchicine and prednisone were slightly more expensive at lower accrued utility. Anakinra was the most expensive anti-inflammatory medication. Anakinra did yield higher utility than naproxen, but at an ICER of €818,504 comparing anakinra to naproxen, both combined with febuxostat would not be considered cost-effective in the Netherlands with WTP-thresholds ranging from €10,000 to €80,000.

The small differences in QALYs gained between flare medications (anakinra, colchicine, naproxen, and prednisone) and higher incremental costs of anakinra over the other medications were also reflected in the cost-effectiveness acceptability curves (CEAC) (**Fig 2**). Naproxen had the highest probability of being cost-effective across the full range of WTP-thresholds, ranging around 33%. As the WTP-threshold increases to €80,000, the probability of naproxen being cost-effective increased to ~42%. However, both colchicine and prednisone had only slightly lower probabilities of being cost-effective than naproxen. Colchicine is stable across the WTP-threshold range at 33% chance of being cost-effective. Prednisone had a lower probability of being cost-effective and it decreased as the WTP-threshold increases. **Table 3** also shows results when flare medication is combined with allopurinol and No ULT. In both cases, naproxen is the favourable option.

**Table 3:** Probabilistic sensitivity analysis for anti-inflammatory treatment.

	Costs (€)	Effect (QALY)	ΔC	ΔE	ICER
Allopurinol					
Naproxen	4,051.37	0.812493	-	-	-
Colchicine	4,066.48	0.812356	15.11	-0.00014	Dominated
Prednisone	4,074.27	0.812356	22.9	-0.00014	Dominated
Anakinra	4,299.31	0.81274	247.94	0.00025	1,003,805.67
Febuxostat					
Naproxen	4,404.72	0.82288	-	-	-
Colchicine	4,426.91	0.82274	22.19	-0.00014	Dominated
Prednisone	4,424.72	0.82277	20.00	-0.00011	Dominated
Anakinra	4,651.09	0.82318	246.37	0.00030	818,504.98
No ULT					
Naproxen	4,012.99	0.788082			-
Colchicine	4,039.99	0.787918	27.00	-0.00016	Dominated
Prednisone	4,040.82	0.757973	27.83	-0.03011	Dominated
Anakinra	4,297.02	0.788356	284.03	0.00027	1,036,605.84

QALY = Quality adjusted life years; ICER = incremental cost-effectiveness ratio; ULT = urate lowering therapy

*ICER for allopurinol vs NO ULT

** ICER for febuxostat vs allopurinol.

### Scenario analyses

In the scenario of patients without flare upon entry, flares per year were lower compared to the base case analysis. This resulted in slightly higher utility and lower costs, but the conclusion remained the same. The second scenario concerns patients with a higher daily probability of starting a flare. Overall this resulted in lower utility and higher costs. Again, the implications did not change. Naproxen remained the most favourable option. The preferred ULT option still depends on the WTP-threshold that would be set in gout, but it remained clear that no ULT was not a preferable option (See S2 Table).

## Discussion

A health economic model was developed for evaluating the costs and effects related to gout treatment strategies that simultaneously covers anti-inflammatory agents for gout flares (i.e. colchicine, naproxen, prednisone, anakinra) and ULT options for hyperuricemia (i.e. allopurinol, febuxostat, or no ULT).

The results of our comparison of ULT strategies suggest that strategies in which no ULT is used would not be considered cost-effective at any WTP threshold that is customary in the Netherlands. This finding supports, from a health-economic point of view, the 2016 updated EULAR guidelines, which was the first to emphasize that ULT should be considered and discussed with every patient from the first presentation of gout with a definite diagnosis [6]. It should be noted that due to the relatively small incremental QALYs between strategies, costs play a rather significant role in the outcome of these analyses. Our results further show that which specific ULT yields the highest net benefit depends on the WTP threshold. In the Netherlands, the WTP threshold ranges from €10,000 to €80,000 and depends on the ‘burden of disease’, estimated using the proportional shortfall method. The ICER of febuxostat compared to allopurinol is €25,173.06, and is thus quite close to the WTP-threshold set for the lowest disease burden category, which is up to €20,000 euro per QALY [15]. Although not yet explicitly defined, the disease burden of the population of gout patients considered in this study could be expected to fall in the lowest category defined by the National Healthcare Institute [15] due to its episodic pattern with longer periods of no attacks. However, there are various methods of calculating burden of disease [1]. With respect to the cost-effectiveness of different ULT, this would indicate that using allopurinol is the preferable option. However, the disease burden of gout varies substantially with severity. For example health-related quality of life of patients with difficult to treat, chronic gout was found to be similar to that of patients with active rheumatoid arthritis [21]. Since the proportional shortfall weighted burden of rheumatoid arthritis corresponds to the highest disease burden category [22], a WTP threshold of €80,000 might be applied to the population of patients with severe gout. This would suggest that febuxostat may be preferable to allopurinol in the treatment of chronic gout. However, as the results of our study were not based on patients with severe gout, this would need to be investigated further in future studies.

The cost-effectiveness of various ULT monotherapies has been compared in several previous, model-based studies [10–12]. In all cases, these studies considered ULT only and, either did not consider the impact of flares on quality of life or used a disutility to account for flares. The current paper specifically focusses on combinations of ULT and flare medication. Furthermore, utility and costs weights were attached to various SUA level related health states. By contrast, in our model utility and costs are mainly determined by the current level of pain experienced by the patient, with a disutility for patients not reaching the SUA target. Utility was mainly determined by flare duration and intensity. This choice was motivated by the consistent findings in previous studies that pain is strongly related to health-related quality of life of gout patients, whereas mixed findings were reported with respect to the relationship between SUA levels and quality of life [23]. The assumption that lowering SUA levels would produce utility gains independently of gout flares was also considered implausible in a recent NICE single technology appraisal of cost-effectiveness evidence in favour of febuxostat, since gout is usually asymptomatic in between flares [24]. Nevertheless, it is interesting to note that despite these differences in model structure, all three studies found results roughly consistent with ours in that applying any ULT was found to be cost-effective relative to no ULT. Additionally, model input was limited by data availability. For example, higher dose allopurinol treatments are rarely administered in daily clinical practice due to intolerance and has not provided an adequate amount of data. It can be hoped for that with the upswing of registries and technological advancements more knowledge and daily clinical practice data becomes available. Furthermore, febuxostat was consistently associated with both higher costs and higher effectiveness compared with allopurinol in the previous studies. Several more studies evaluated cost-effectiveness of various ULT sequences [25–27]. However, our analyses solely focused on monotherapies since this has been shown to be the most common treatment pattern in clinical practice [12, 28]. It should be noted though that various international guidelines currently recommend titrating allopurinol dosages up to 900 mg/day. It seems likely that a higher percentage of patients would be able to reach the SUA target at higher allopurinol dosages. Unfortunately, no suitable data was identified in our literature review to be able to assess this treatment strategy in our model.

To the best of our knowledge, the current study is also the first model-based study to examine cost-effectiveness of anti-inflammatory treatments for gout attacks. Results of our study reveal that naproxen was the favourable treatment at any WTP-threshold, in combination with any of the ULT, although overall differences in cost-effectiveness between conventional treatment strategies remained small. In addition, our results showed that treatment with anakinra, although accruing slightly higher health outcomes after one year compared to conventional treatments, was not cost-effective, primarily driven by its high costs per treatment. Costs over one year for strategies including anakinra were approximately €200,- higher than the other gout flare medications. Although this is a smaller difference than what the difference in absolute drug prices between anakinra and, for example, naproxen would suggest, our findings do not support a role for anakinra as a first line treatment in the overall gout population.

The current study had some limitations. First, the amount of data used to estimate the pain transition probabilities for each gout flare treatment option, was limited. This resulted from the need to have access to patient level data to populate the model. In particular, data with regard to anakinra and colchicine were based on a single randomized controlled trial. The resulting uncertainty about the relative effectiveness of the different treatments may have undermined our ability to differentiate the efficacy of different anti-inflammatory treatments. Second, the occurrence of (serious) adverse events and their associated costs and consequence on utilities, were not included in the model. This also applies for using prophylaxis when initiation ULT as recommended by gout guidelines. Next, insufficient data was available to consider running a longer time horizon for this model. Information on efficacy and safety of gout treatments in the longer term was available, however, for using that data in modelling studies like the current paper it would have to have been linked to quality of life or utility data, which was not an options with the current existing data. The one-year time horizon for this model allowed to focus on the effects of gout in a newly diagnosed population. For patients that experience relatively many flares, quality of life mostly depends on their health states (and utility) during those flares. While a longer term model would certainly also be interesting and necessary for decision makers, long-term effects are not within the scope of this model. A longer time horizon would mean that patients are more stable on their ULT, experience less flares and may choose to discontinue or stop ULT and rarely need flare medication. The scope of the current research has specific attention for utilities and costs during flares, therefore the time horizon has been limited. A longer horizon would demand a different focal point. When looking at a longer term model, several more events could be included. In an ideal situation with a richness of datasources and unlimited modelling options, it would be fascinating to be able to include SAE’s, ULT sequences, medication discontinuation, and other long-term gout events. Insufficient data was available for us to consider the IL-1 inhibitor, canakinumab, for the treatment of gout flares, or second-line ULT agents as pegloticase and lesinurad, which has just recently been approved by the Food and Drug Administration in combination with allopurinol [29]. However, none of these drugs are likely to become first line treatment options for gout and hyperuricemia in the near future.

## Conclusions

In conclusion, the findings of our study show that ULT, with either allopurinol or febuxostat, are cost-effective first-line ULT agents for treating hyperuricemia. For the treatment of gout flares, conventional first-line treatments (i.e. colchicine, naproxen, prednisone) had similar health economic implications, of which naproxen had the most favourable costs and effects profile.

## Supporting information

S1 File Cheers checklist. (DOCX)

S1 Table Transition matrix for naproxen treatment. (DOCX)

S2 Table PSA scenario analyses. QALY = Quality adjusted life years; ICER = incremental cost-effectiveness ratio; ULT = urate lowering therapy; *ICER for allopurinol vs No ULT; ** ICER for febuxostat vs allopurinol.
(DOCX)
