# Supplementary material for: Model-based cost-effectiveness analyses comparing combinations of urate lowering therapy and anti-inflammatory treatment in gout patients
Source: PLoS One. 2022 Jan 28;17(1):e0261940. doi: 10.1371/journal.pone.0261940 (PMC8797232; doi:10.1371/journal.pone.0261940)
Supplement: S1 Table — (DOCX) [file pone.0261940.s002.docx]

**S1 Table. Transition Matrix for Naproxen Treatment**

*Transition matrix for naproxen*

|  | End pain state (%) | | | |
| --- | --- | --- | --- | --- |
| Start pain state | No | Mild | Moderate | severe |
| No | 91.46 | 6.09 | 1.21 | 1.21 |
| Mild | 13.28 | 80.44 | 4.42 | 1.85 |
| Moderate | 0.87 | 45.61 | 43.86 | 9.65 |
| Severe | 2.17 | 14.13 | 36.96 | 46.74 |
