# Supplementary material for: Model-based cost-effectiveness analyses comparing combinations of urate lowering therapy and anti-inflammatory treatment in gout patients
Source: PLoS One. 2022 Jan 28;17(1):e0261940. doi: 10.1371/journal.pone.0261940 (PMC8797232; doi:10.1371/journal.pone.0261940)
Supplement: S2 Table — QALY = Quality adjusted life years; ICER = incremental cost-effectiveness ratio; ULT = urate lowering therapy; *ICER for allopurinol vs No ULT; ** ICER for febuxostat vs allopurinol. (DOCX) [file pone.0261940.s003.docx]

**S2 Table. PSA Scenario analyses**

*Table 2: No flare upon entry full results*

|  | Costs (€) | Effects (QALY) | ΔC (€) | ΔE (QALY) | ICER (ΔC/ΔE) |
| --- | --- | --- | --- | --- | --- |
| Febuxostat | | | | | |
| Naproxen | 4,146 | 0.82484 | - | - | - |
| Colchicine | 4,152 | 0.82481 | 5.95 | -0.00003 | Dominated |
| Prednisone | 4,147 | 0.82481 | 0.42 | -0.00003 | Dominated |
| Anakinra | 4,217 | 0.82482 | 71.19 | -0.00002 | Dominated |
| Allopurinol | | | | | |
| Naproxen | 3,561 | 0.82436 | - | - | - |
| Colchicine | 3,566 | 0.82426 | 4.77 | -0.00010 | Dominated |
| Prednisone | 3,563 | 0.82431 | 1.54 | -0.00005 | Dominated |
| Anakinra | 3,643 | 0.82431 | 81.70 | -0.00005 | Dominated |
| No ULT | | | | | |
| Naproxen | 3,885 | 0.78639 | - | - | - |
| Colchicine | 3,894 | 0.78632 | 8.49 | -0.00007 | Dominated |
| Prednisone | 3,893 | 0.78634 | 7.67 | -0.00005 | Dominated |
| Anakinra | 3,989 | 0.78636 | 103.91 | -0.00003 | Dominated |
| ULT comparison | | | | | |
| No ULT + Naproxen | 3,885 | 0.78639 |  |  |  |
| Allopurinol + Naproxen | 3,561 | 0.82436 | -324 | 0.03797 | Dominating* |
| Febuxostat + Naproxen | 4,146 | 0.82484 | 585 | 0.00048 | 1,218,750** |

QALY = Quality adjusted life years; ICER = incremental cost-effectiveness ratio; ULT = urate lowering therapy; *ICER for allopurinol vs No ULT; ** ICER for febuxostat vs allopurinol

*Table 3: Increase flare chance full results*

|  | Costs (€) | Effects (QALY) | ΔC (€) | ΔE (QALY) | ICER (ΔC/ΔE) |
| --- | --- | --- | --- | --- | --- |
| Febuxostat | | | | | |
| Naproxen | 4,389 | 0.82507 | - | - | - |
| Colchicine | 4,417 | 0.82492 | 28.25 | -0.00015 | Dominated |
| Prednisone | 4,406 | 0.82493 | 17.05 | -0.00014 | Dominated |
| Anakinra | 4,642 | 0.82535 | 252.85 | 0.00027 | 927,118 |
| Allopurinol | | | | | |
| Naproxen | 4,069 | 0.80843 | - | - | - |
| Colchicine | 4,099 | 0.80829 | 29.22 | -0.00013 | Dominated |
| Prednisone | 4,090 | 0.80828 | 20.23 | -0.00014 | Dominated |
| Anakinra | 4,336 | 0.80872 | 266.45 | 0.00030 | 896,226 |
| No ULT | | | | | |
| Naproxen | 4,043 | 0.78905 | - | - | - |
| Colchicine | 4,075 | 0.78889 | 32.34 | -0.00015 | Dominated |
| Prednisone | 4,076 | 0.78890 | 33.02 | -0.00015 | Dominated |
| Anakinra | 4,331 | 0.78934 | 288.38 | 0.00029 | 978,458 |
| ULT Comparison | | | | | |
| No ULT + naproxen | 4,043 | 0.78905 | - | - | - |
| Allopurinol + naproxen | 4,069 | 0.80843 | 26 | 0.01938 | 1,342* |
| Febuxostat + naproxen | 4,389 | 0.82507 | 320 | 0.01664 | 19,231** |

QALY = Quality adjusted life years; ICER = incremental cost-effectiveness ratio; ULT = urate lowering therapy; *ICER for allopurinol vs No ULT; ** ICER for febuxostat vs allopurinol
